# Tespa1 plays a role in the modulation of airway hyperreactivity through the IL-4/STAT6 pathway

**DOI:** 10.1186/s12967-020-02621-4

**Published:** 2020-11-23

**Authors:** Ruhui Yang, Guangli Wang, Lingyun Li, Hanjiang He, Mingzhu Zheng, Linrong Lu, Songquan Wu

**Affiliations:** 1 Department of Pharmacology, College of Medicine and Health, Lishui University, No. 1 Xueyuan Road, Liandu District, Lishui, 323000 China; 2 College of Medicine and Health, Lishui University, No. 1 Xueyuan Road, Liandu District, Lishui, 323000 China; 3 Clinical Laboratory, Lishui People’s Hospital, Lishui, 323000 China; 4 Program in Molecular and Cellular Biology, Zhejiang University School of Medicine, Hangzhou, 310058 China

**Keywords:** Tespa1, Asthma, Mast cell, STAT6

## Abstract

**Background:**

Thymocyte-expressed, positive selection-associated 1 (Tespa1) is a critical signaling molecule in thymocyte development. This study aimed to investigate the regulatory effect of Tespa1 on mast cells in the pathogenesis of asthma and its relationship with the interleukin (IL)-4/signal transducers and activators of transcription 6 (STAT6) signaling pathway.

**Methods:**

Tespa1 mRNA expression analysis and IgE levels were carried out using the induced sputum of 33 adults with stable asthma and 36 healthy controls. *Tespa1-*knockout mice (*Tespa1*^−/−^, KO) and C57BL/6 background (wild-type, WT) mice were sensitized and treated with ovalbumin (OVA) to establish an asthma model. Pathological changes, number and activity of mast cells, and changes in activation of the IL-4/STAT6 pathway in lung tissue were detected. The changes of tryptase expression and STAT6 activation after mast cell gene knockout were analyzed in vitro. The changes of enzyme expression and STAT6 activation after mast cell gene knockout were analyzed in vitro. The association between the Tespa1 and p-STAT6 was analyzed by co-immunoprecipitation method.

**Results:**

Compared with the healthy controls, *Tespa1* expression was decreased, and IgE levels were elevated in the sputum of asthmatic patients. Animal experiments showed that *Tespa1*^*−/−*^ mice exhibited more severe inflammation, higher quantity of goblet cells and mast cells in the bronchium, and greater expression of mast cell tryptase, which is induced by ovalbumin, than WT mice. And IL-4, IL-13, phospho-Janus kinase 1, and p-STAT6 expressions presented a higher increase in the *Tespa1*^*−/−*^ mouse model than in the WT mouse model. Further in vitro studies confirmed that IL-4 could more significantly promote tryptase and p-STAT6 activities in *Tespa1*^−/−^ mast cells than their WT counterparts. Correlation analysis results showed a negative correlation between Tespa1 and p-STAT6. Co-immunoprecipitation results demonstrated an association between Tespa1 and p-STAT6.

**Conclusions:**

Altogether, our results indicate that Tespa1 can negatively regulate mast cell activity, and this event is related to the mast cell IL-4/STAT6 signaling pathway and could be therapeutically exploited to treat asthma attacks.

## Background

Mast cells play an important role in the innate and adaptive immunities related to the pathophysiologic process of asthma. Mast cells not only act as effector cells during allergic reactions, but also perform a complex role in the induction and regulation of adaptive immune responses of asthma [1–3]. When an allergen enters the atopic patient, IgE is specifically synthesized by B lymphocytes and bind to the high-affinity IgE receptor (FcεRI) on the surface of mast cells and basophils [4, 5]. If the allergen re-enters the body, it can bind with IgE FcεRI on the mast cells, thus promoting cell activation, synthesizing and releasing various active mediators, such mediators as histamine, chemokine, neutrophil chemokine, prostaglandin [6, 7]. These mediators and cytokines play a pivotal role in the pathogenesis of asthma and inflammation. Interleukin (IL)-4 and IL-13 are among the cytokines produced by mast cells. IL-4 is the first cytokine shown to be produced by mast cells. IL-4 production in mast cells has been studied in relation to IgE-mediated activation [8, 9]. IL-4 is a multifunctional cytokine that plays an important role in inducing allergic Th2-type immune responses [10]. IL-4 activates signal transducers and activators of transcription 6 (STAT6) and induces the expression of IL-4—inducible genes, including class II major histocompatibility molecules, low-affinity IgE receptor, and IL-13 [9, 11]. Therefore, IL-4/STAT6 signaling plays a central role in the participation of mast cells in asthma progression.

Thymocyte-expressed, positive selection-associated 1 (Tespa1) is a critical signalling molecule in thymocyte development [12]. Tespa1 is highly expressed in mast cells and is involved in the negative regulation of mast cell activation and mediation of allergic reactions by negatively regulating FcεRI-mediated signaling. In mast cells, Tespa1 works as an adjusting lever to determine the allocation of signaling molecules to either the LAT1 (linker for activation of T cells family, member 1) or the LAT2. The preferential assembly of the LAT2 promoted by Tespa1 might serve as a reservoir to regulate the signal strength of LAT1. IP3R1 (inositol 1,4,5-trisphosphate receptor type 1) can be recruited into the LAT2 complex by Tespa1 and then dephosphorylated to inhibit the activity [13, 14]. However, the role of Tespa1 in the IL-4/STAT6 signaling pathway, an important pathway for mast cell function and asthma pathogenesis, remains unclear. In this study, we observed that Tespa1 is associated negatively with the IL-4/STAT6 signaling pathway in the activation of mast cells, thus providing a new intervention target for mast cells in the pathogenesis of asthma.

## Materials and methods

### Detection of Tespa1 mRNA expression in the sputum of asthma patients and healthy controls

33 patients with physician diagnosed asthma and 36 healthy adults without asthma were enrolled for this study. All subjects were non-smokers, and those on maintenance oral corticosteroid therapy were excluded [15]. All subjects were recruited from the Respiratory Clinic of Lishui People’s Hospital, Lishui, China. The Global Initiative for Asthma guidelines (www.ginasthma.org) were used by a respiratory medicine specialist to diagnose asthma. Total RNA was extracted from the induced sputum samples asthmatic patients and healthy controls by using the TRIzol reagent (Invitrogen, USA) according to the manufacturer’s instructions. All sputum samples were processed with RNAprotect cell reagent and phosphate-buffered saline (PBS) according to the manufacturer’s instructions. An IQ SYBR Green SuperMix polymerase chain reaction (PCR) array kit was purchased from Bio-Rad (USA). Two micrograms of extracted RNA was converted to cDNA by Moloney murine leukemia virus reverse transcriptase (Fermentas, CAN), which was used according to the manufacturer’s instructions. The cDNA was amplified using the following forward and reverse primers: forward: 5′-CAACCATCCAACT GATGTGCC-3′ and reverse: 5′-TCCAACACAA CTTGGTCCAAA′; for β-actin, forward: 5′-TGACGTGGACATCCGCAAAG-3′ and reverse: 5′-CTGGAAGGTG GACAGCGAGG-3′. The human β-actin housekeeping gene was used as an internal control. The primers were designed and synthesized at Shanghai Generay Biotech (Shanghai, China). The reaction was evaluated using a CFX Connect Real-Time PCR system (Bio-Rad, USA). The relative expression levels of the mRNA in each sample were calculated by normalizing the threshold cycle (Ct) value to the Ct value of the β-actin housekeeping gene by using the 2^−ΔΔCt^ method. The mRNA expression levels were expressed in arbitrary units.

### IgE enzyme-linked immunosorbent assay (ELISA)

The sputum was collected in a plastic container, and homogenized by adding an equal volume of 1% dithiothreitol for 30 min at room temperature. After incubation, the supernatant was centrifuged at 2000 r/min for 10 min, and the sputum IgE levels were tested using the Human IgE ELISA Kit (EK175-96, MultiSciences, China) as previously described [16]. Samples measurements were obtained at 450 nm by using a SpectraMax Plus 384 microplate reader (MD, USA) and SoftMax Pro software.

### Animal and mouse models of acute asthma

*Tespa1-*knockout mice (*Tespa1*^−/−^, KO) and C57BL/6 background (wild-type, WT) mice were generated via homologous recombination-mediated gene targeting at the Shanghai Research Center for Model Organisms as previously described [13]. The mice were housed in a temperature-controlled room under a 12 h dark/light cycle and were allowed access to food and water ad libitum. This study was conducted in strict accordance with the recommendations of the Guide for the Care and Use of Laboratory Animals of the National Institutes of Health, and its protocol was approved by the Animal Research Ethics Board of the Lishui University (Lishui, Zhejiang Province, China. Permit Number: 0901-2018).

*Tespa1*^−/−^ mice and WT mice were divided into two groups (15 mice per group), namely, the control and asthma groups. The asthma mouse model was established using a traditional protocol [17, 18]. Briefly, allergic asthmatic reactions and airway remodelling were induced in the abovementioned mice by using chick ovalbumin (OVA, Sigma, USA). Specifically, the mice were initially sensitized through intraperitoneal (i.p.) injections of PBS with 25 μg OVA in 1 mg aluminium hydroxide gel (Thermo Scientific Inc., Germany) and in 0.2 mL PBS, at pH 7.4, on days 0, 7, and 14. The mice were subsequently randomized into groups that were repeatedly administered nebulized with 5% OVA in PBS or PBS alone by using an ultra-sonic nebulizer with an aerosol chamber (Yuyue Medical Equipment & Supply Co., Ltd., Shanghai, China) on days 15 to 42. The mice were given aerosolized OVA for 30 min each day for 28 consecutive days. The mice in the control group were i.p. and atomized with equal volume of PBS.

### Measurement of airway resistance

On day 42, nine mice from each group were anesthetized via i.p. injections of 300 mL pentobarbital sodium (60 mg/kg) before undergoing tracheostomy tube insertion. Airway resistance and compliance measurements were performed using a FinePointe RC system (Buxco Research Systems, Wilmington, NC). The mice were subsequently challenged with aerosolized PBS (baseline) acetylcholine treatment doses of 0, 1, 2, 4, 8, and 16 mg/mL. Average compliance values were recorded during a 3 min period following each challenge [19].

### Measurement of IL-4 and IL-13 protein expressions by ELISA

Broncio Alveolar Lavage (BAL) was centrifuged at 1000×*g* for 5 min at 4 °C. After centrifugation, the IL-4 and IL-13 protein expression levels in the BAL supernatant were measured using a sandwich ELISA kit (70-EK204, 70-EK213, MultiSciences, China) according to the manufacturer’s instructions. Samples measurements were obtained at 450 nm by using a SpectraMax Plus 384 microplate reader (MD, USA) and SoftMax Pro software.

### Lung histology

For the histological evaluation of mouse lung tissue specimens, we fixed the left lung of each mouse in 10% buffered formalin. The fragments were then dehydrated, cleared, and embedded in paraffin. The whole lung was serially sectioned (3–4 μm-thick), stained with H&E for pathological analysis, and stained with periodic acid-Schiff (PAS) for goblet cell detection. The degree of peribronchial and perivascular inflammation was evaluated according to a subjective scale ranging from 0 to 4 [20, 21]. The degree of cell infiltration in the above tissues was scored as follows: 0, no cells; 1, a few cells; 2, a ring of cells with a depth of one cell; 3, a ring of cells with a depth of two to four cells; 4, a ring of cells with a depth of more than four cells. Reticular basement membrane thickness was measured by image analysis of multiple randomly selected tissue sections, with each of section comprising 30 analysis points, by using an Olympus software microscope system. Repeat measurement error was assessed by performing multiple measurements on a single membrane area in four subjects, as previously described [22].

The degree of goblet cell hyperplasia in the airway epithelium was quantified in accordance with the following five-point system: 0, no goblet cells; 1, < 25% of the cells in the epithelium are hyperplasic; 2, 25–50% of the cells in the epithelium are hyperplastic; 3, 50–75% of the cells in the epithelium are hyperplastic; and 4, > 75% of the cells in the epithelium are hyperplastic. Five randomly distributed left lung airway sections were analyzed in each mouse, and the average score was calculated by summing the scores from the five fields.

Mast cells were stained with toluidine blue. The sections were stained with 1% toluidine blue (Sigma-Aldrich) solution in 1% sodium chloride with diluted 1:10 for 10 min, washed for 1 min, differentiated with 0.5% glacial acetic acid for several seconds, and washed for 5 min. Mast cells were identified by metachromatic staining of their granules. The stained slides were all quantified under identical light microscope conditions, in terms of magnification, gain, camera position, and background illumination [23, 24].

### Immunofluorescence and horseradish peroxidase (HRP)-diaminobenzidine immunohistology

For immunofluorescent staining, sections of lungs or cells were incubated with the antibody against mast cell tryptase (ab2378, Abcam, UK), Tespa1 (R1309-16, HuaAn Biotechnology, China), p-STAT6 (ab263947, Abcam, UK) and DAPI (4ʹ,6-diamidino-2-phenylindole, Life Technologies,), and images were obtained by using a confocal laser scanning microscope (LSM 880, Zeiss). The protein expression levels were analyzed using Image J.1.44 software.

For immunohistochemical staining, the slides were incubated with 3% H_2_O_2_ for 10 min after dewaxing, and then washed with PBS for 5 min at room temperature. Antigen retrieval was performed in citrate buffer (pH 6.0) by microwave heating, and blocking was performed with 10% non-immune goat serum for 30 min after cooling. The slides were incubated with an antibody against mast cell tryptase (ab2378, Abcam, UK) overnight at 4 ℃. After rinsing with PBS, the sections were incubated with the HRP-conjugated secondary antibody (Maixin, Fuzhou, China) for 30 min at room temperature. Hematoxylin was applied as a counterstain. Eight fields were randomly selected for the quantification of positive cells in every sample, as previously described [19].

### Western blot analysis

Proteins of lungs tissues were extracted with RIPA buffer [50 mM Tris (pH 8.0), 150 mM NaCl, 1 mM ethylenediaminetetraacetic acid disodium, 10% glycerol, 2% Triton X-100, and a protein inhibitor mixture (Beyotime Biotechnology, Shanghai, China)]. For immunoblot analysis, 30 μg solubilized protein was loaded and resolved by sodium dodecyl sulphate polyacrylamide gel electrophoresis on 8–15% gels. 6 samples were randomly selected from each group for Western blot detection. To detect the expression of one protein, 24 samples were divided into two gels, and the experiment was carried out simultaneously under same conditions. The proteins were then transferred to polyvinylidene difluoride (PVDF) membranes, blocked with 5% skim milk in PBS/Tween-20 for 1 h, and incubated with primary antibodies targeting phospho-Janus kinase 1 (p-JAK1) (D7N4Z, Cell signaling Technology), p-JAK2 (3771, Cell signaling Technology), p-STAT6 (ab263947, Abcam) and GAPDH (MAB5465, MultiSciences Biotech) overnight at 4 °C. Membranes were then washed and incubated with secondary HRP-labelled anti-rabbit/anti-mouse antibodies (1:5000). Chemiluminescent images of the blots were captured using a ChemiDoc System. Image J software was used to calculate the integrated absorbance (IA) of the identified bands, and the expression of each protein was calculated using the following formula: Relative protein expression = IA_protein_/IA_GAPDH_ [25].

### Primary pulmonary mast cell culture and treatment

Primary pulmonary mast cells were derived from the lungs of 4-week-old mice as previously described [26, 27]. In brief, lung samples were cut into pieces, dissociated by collagenase (50 U/mL in Hanks’ balanced salt solution), and filtered through a 40 μm filter. The cells were cultured in Dulbecco’s modified medium (Gibco Invitrogen, USA) containing 10% fetal bovine serum (Gibco). Mast cells were confirmed via flow cytometric analysis of surface markers, CD117 (553869; BD, CA) and FcεRI (11-5898; eBioscience, CA). The cells were seeded in six-well plates at a density of 1 × 10^6^ cells/mL, and 20 ng/mL IL-4 (R&D Systems) was simultaneously applied for 1 and 4 h. Then the cells were collected for testing [28, 29].

### Co-immunoprecipitation

Immunoblotting was performed as previously described [30, 31]. The cells were harvested in a cell lysis buffer (10 mM Tris–HCl, pH 7.5, 150 mM NaCl, 10 mM MgCl_2_, 0.5% Triton X-100, 10 mM dithiothreitol, and protease/phosphatase inhibitor cocktails). Cell extracts were then incubated with 2 μg p-STAT6 antibody and 20 μL LProteinA/G at 4 ℃ for 3 h. After the immunoprecipitation reaction, the samples were centrifuged at 4 ℃ 3000 rpm for 5 min, and then wash thrice. Finally, the proteins were eluted with elution buffer and boiled for 5 min prior to Western blot analysis. The PVDF membranes were blocked with a solution containing primary antibodies against Tespa1. A control lacking the primary antibodies was incubated with anti-mouse IgG.

### Statistical analysis

The data are reported as the mean ± SD. Statistical significance was determined by ANOVA followed by Tukey’s correction for multiple comparisons or Student’s two-tailed *t* test for independent means. Non-parametric analyses were performed using Kruskal–Wallis one-way analysis. Pearson correlation (*r*) was used for correlation analysis. All analyses were performed using SPSS 11.0 for Windows (SPSS) software*. P* values less than 0.05 were considered statistically significant.

## Results

### The expression level of Tespa1 mRNA in the sputum of asthmatic patients was lower and IgE level was higher than that of healthy controls

**Fig. 1:**
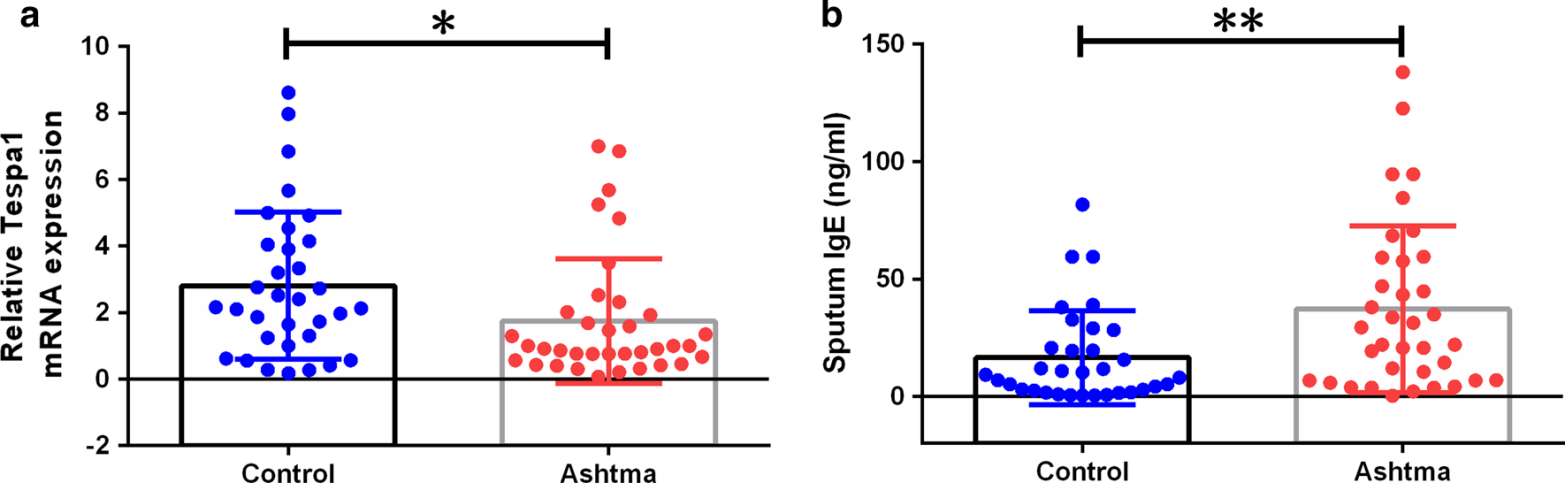
Tespa1 mRNA expression and IgE content in sputum of asthmatic and normal individuals. **a** The relative mRNA expression level of Tespa1 expressed as the ratio of the mRNA level of the target gene to the mRNA levels of theβ-actin gene in sputum. **b** IgE levels in sputum of asthmatic and normal individuals. Results are expressed as mean ± SD. Control: healthy people (*n* = 36). Asthma: patients with asthma (*n* = 33). *P < 0.05 between groups. **P < 0.01 between groups

To detect the role of Tespa1 in the pathogenesis of asthma patients, we selected sputum from 33 patients with chronic asthma and 36 healthy individuals to test the expression of Tespa1 mRNA and IgE levels. Results showed that compared with the healthy people, the level of Tespa1 mRNA expression of asthma patients was relatively lower, and IgE levels in the sputum of the patients were significantly increased (*P* < 0.05) (Fig. 1).

### OVA-induced asthma is more serious in Tespa1^−/−^mice than in WT mice

**Fig. 2:**
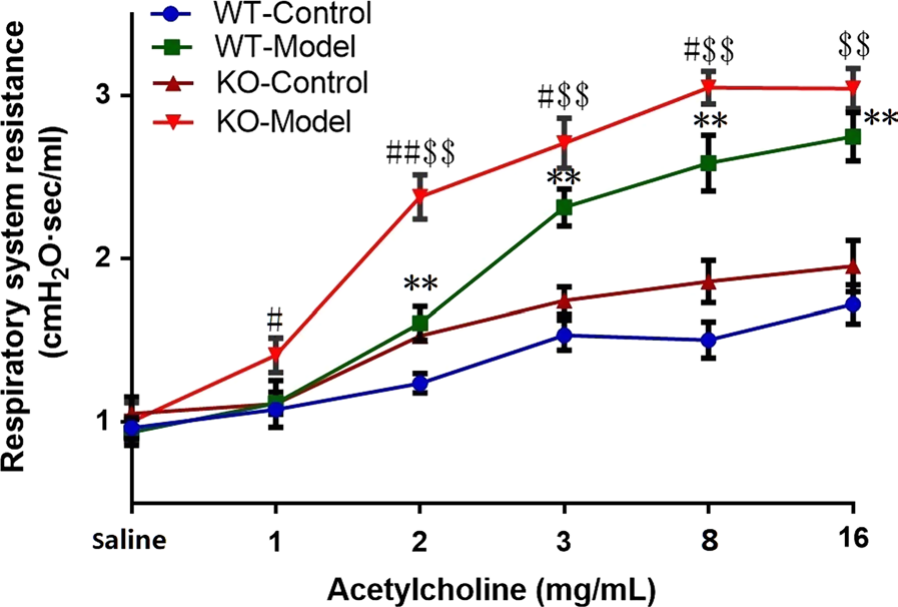
Effect of *Tespa1* on acetylcholine-induced airway hyperresponsiveness (AHR) in asthmatic mice. Airway resistance measurements were performed using a FinePointe RC system. The mice were challenged with aerosolized PBS (baseline) before treatment with acetylcholine at the following ascending doses: 0, 1, 2, 4, 8, and 16 mg/mL. KO: *Tespa1*^−/−^ mice; WT: wild-type mice. The data are expressed as mean ± SD (*n* = 9 per group). **P < 0.01 compared with the WT control group; ^#^*P* < 0.05 compared with the WT model group; ^##^*P* < 0.01 compared with the WT model group. ^$$^*P* < 0.01 compared with the KO control group

**Fig. 3:**
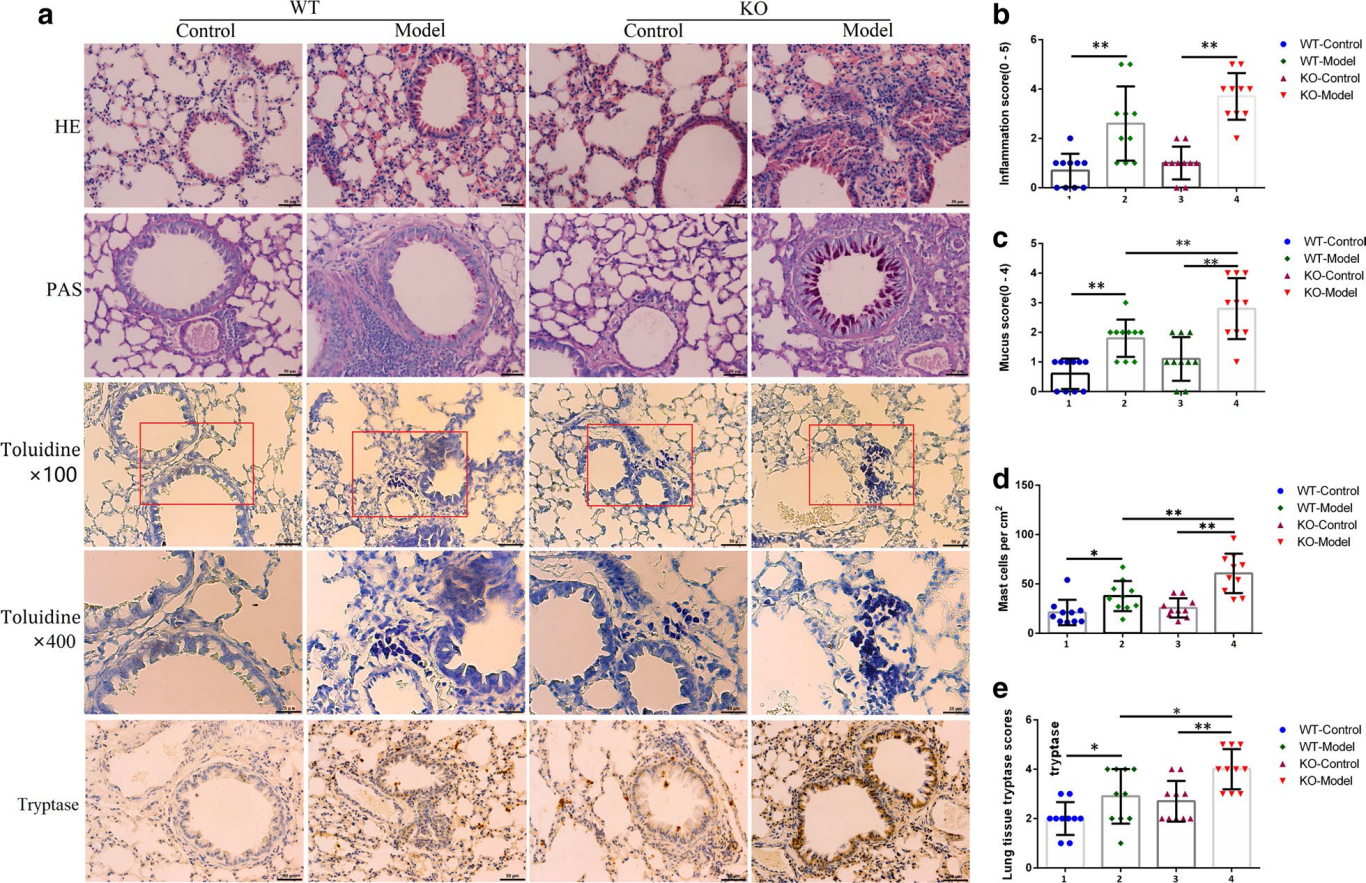
OVA-induced lung histological and tryptase changes in *Tespa1*^−/−^ and WT mice as determined by H&E staining, PAS staining, toluidine blue staining, and immunohistochemistry. **a** Images of H&E staining, PAS staining, toluidine blue staining and immunohistochemistry. **b** Quantitative analysis of the degree of inflammatory cell infiltration in the lung sections, based on the methods developed by Myou and Lee [45, 46]. **c** Kruskal–Wallis one-way analysis of mucus production in the lung sections was performed in accordance with the methods developed by Myou and Lee. **d** Number of mast cells in lung sections. **e** Immunohistochemical scores of tryptase. KO: *Tespa1*^−/−^ mice; WT: wild-type mice. *P < 0.01 between groups. **P < 0.01 between groups. Mean ± SD, *n* = 8–10 mice per group

To further explore the role of Tespa1 in asthma, we assessed the OVA-induced asthma models of *Tespa1*^−/−^ and WT mice. Airway function was assessed by measuring the changes in lung resistance and compliance elicited by acetylcholine inhalation, which induced bronchoconstriction. The airway resistance for each of the four groups was analyzed, and the results are shown in Fig. 2. Airway resistance increased in OVA-primed/challenged mice from the *Tespa1*^−/−^ and WT groups compared with the control mice. Airway resistance increased in *Tespa1*^−/−^ mouse model compared with the WT mice. Pathology results proved the more evident inflammation in *Tespa1*^−/−^ asthma mice than in WT mice. Moreover, PAS dyeing results indicated that the distribution of goblet cells was more intensive in the small bronchial of *Tespa1*^−/−^ asthma mice than that in the WT group (Fig. 3a–c).

### KO of Tespa1 enhanced lung mast cell activity

Further experiments were performed to evaluate the regulatory effect of Tespa1 on mast cells in asthmatic mice. We used toluidine blue staining to evaluate the changes in the number of mast cells in lung tissue, and immunohistochemical staining with tryptase to analyze the changes in mast cell enzyme activity. Five randomly selected digital images were obtained from each lung section using a phase contrast microscope (Olympus BX43, Tokyo, Japan). Two reviewers blinded to the diagnosis counted the numbers of mast cell and tryptase expression score were recorded for each lung section. The results of toluidine blue staining of lung tissues showed that OVA induced an increase in the distribution of mast cells in alveolar parenchyma of mice, and the *Tespa1*^*−/−*^ model mice showed more numerous mast cells compared with that in WT model mice (Fig. 3a, d). The results of immunohistochemical analysis showed that, compared with WT mice, *Tespa1*^*−/−*^ mice had increased expression of tryptase, a marker of mast cell activation (Fig. 3a, e). This might reasonably correlate with an increase of mast cell activity in *Tespa1*-deficient mice.

### KO of Tespa1 can increase the activation of STAT6 pathway in asthma mice

**Fig. 4:**
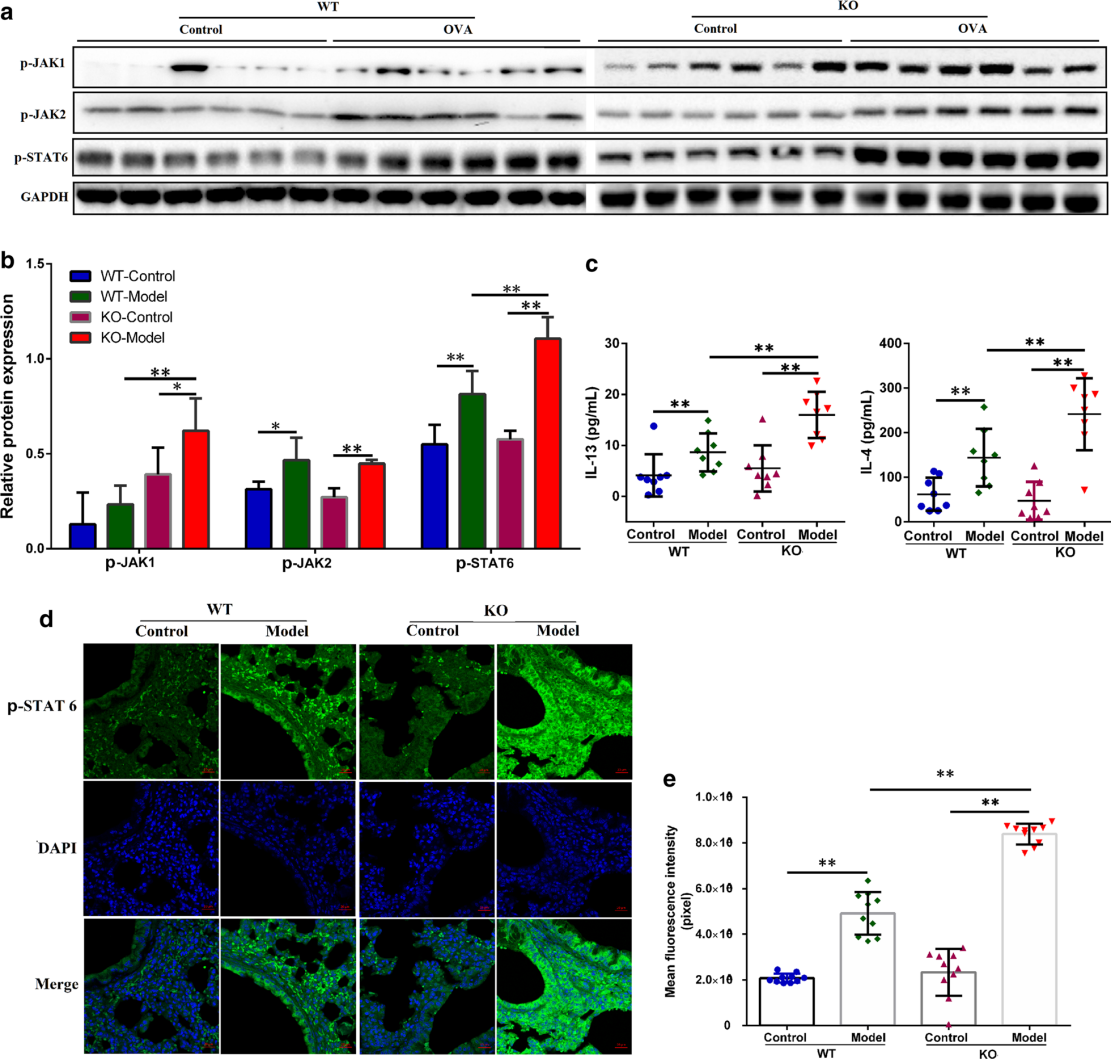
OVA-induced changes in the protein expression of the IL-4/STAT 6 signaling pathway in *Tespa1*^−/−^ and WT mice, as determined by Western blot, ELISA, and immunofluorescence technique. **a** Electrophoretograms of proteins of P-JNK1, P-JNK2, p-STAT6 and GAPDH. **b** Graph of the relative protein expressions of P-JNK1, P-JNK2, and p-STAT6 as determined by Western blot analysis. **c** Quantitative analysis of IL-4 and IL-13 levels in the BAL of mice by ELISA. **d** Immunofluorescence staining for p-STAT6. **e** Table of average p-STAT6 fluorescence intensities. KO: *Tespa1*^−/−^ mice; WT: wild-type mice. **P < 0.01 between groups. *P < 0.05 between groups. Mean ± SD, *n* = 8–10

To investigate the role of Tespa1 in the JAK1/2-STAT6 pathway, proteins from the lung tissues of OVA-induced asthma models of *Tespa1*^−/−^ and WT mice, and control mice were extracted. Six samples were randomly selected from each group, and the expressions of p-JAK1, p-JAK2 and p-STAT6 were detected by Western blot. Due to the large amount of samples, two gels were used for each protein test under the same experimental conditions. Western blot analysis results showed that OVA enhanced p-JAK1, p-JAK2, and p-STAT6 protein expression levels in *Tespa1*^−/−^ and WT mice. p-JAK1 and p-STAT6 protein expression levels significantly increased in the *Tespa1*^−/−^ mouse model compared with the WT model (Fig. 4a, b). To further verify the effect of Tespa1 on the IL4/STAT6 pathway, levels of IL-4 and IL-13 in BAL of OVA-induced asthma models of *Tespa1*^*−/−*^ and WT mice, and control mice were detected by ELISA. The results showed that the *Tespa1*^−/−^ mouse model exhibited increased expressions of IL-4 and IL-13 than the WT mouse model (Fig. 4c). Finally, the changes of p-STAT6 were analyzed by immunofluorescence method to verify the activation of KO on Tespa1 STAT6. And the results showed that OVA could induce the expression of p-STAT6. In *Tespa1*^*−/−*^ mice, the increased expression of p-STAT6 was more remarkable compared with that in WT mice.

### Mast cell activity and IL4/STAT6 pathway are closely related to Tespa1

**Fig. 5:**
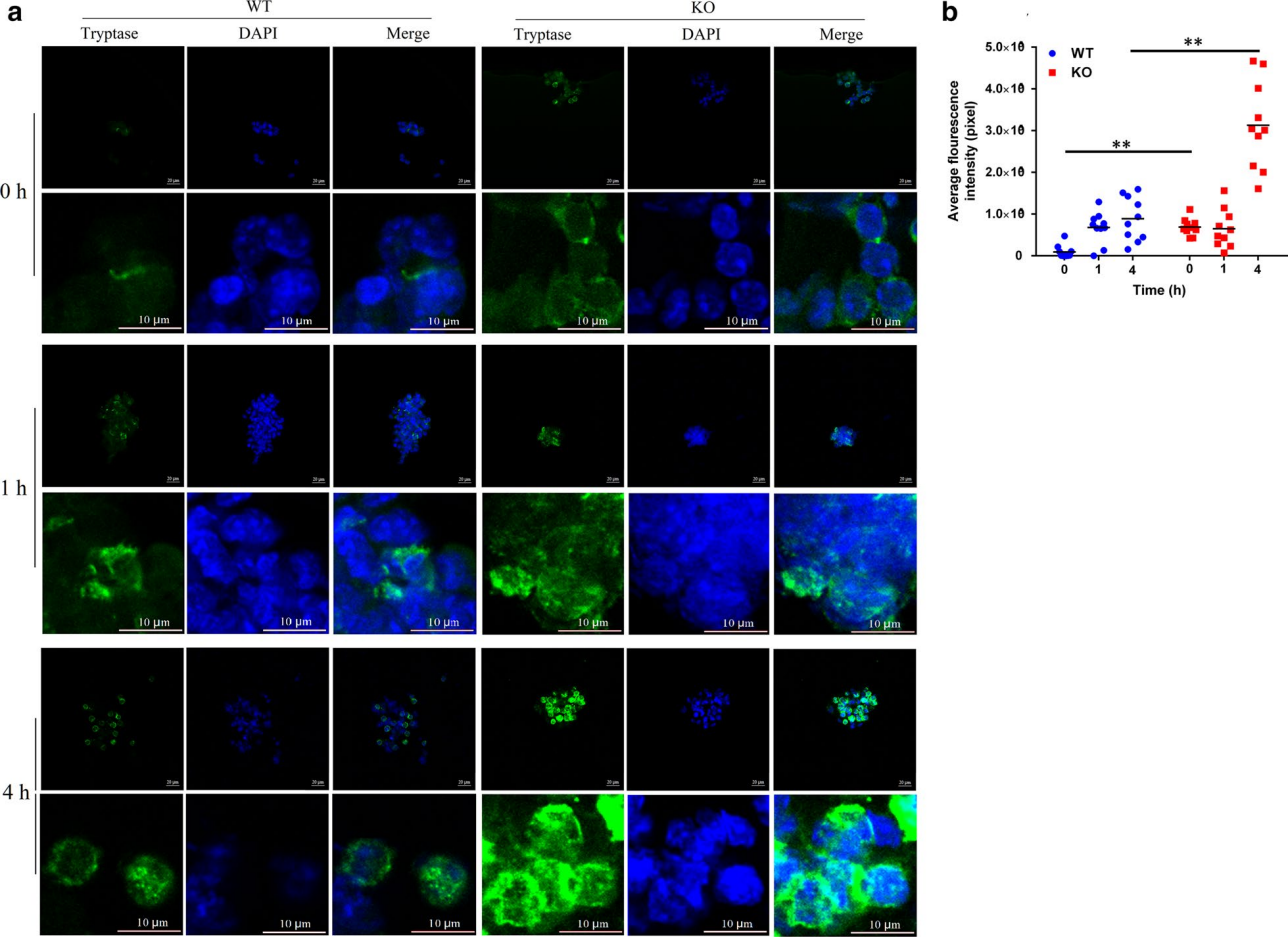
Tryptase expression in the mast cells of *Tespa1*^−/−^ and WT mice after IL-4 stimulation. Primary pulmonary mast cells were derived from the lungs of mice, and IL-4 (20 ng/mL) was simultaneously applied for 1 and 4 h. Immunofluorescence was used to detect tryptase expression. **a** Immunofluorescence staining for p-STAT6. **b** Table of average tryptase fluorescence intensities. KO: *Tespa1*^−/−^ mice; WT: wild-type mice. **P < 0.01 between groups. Mean ± SD, *n* = 10

**Fig. 6:**
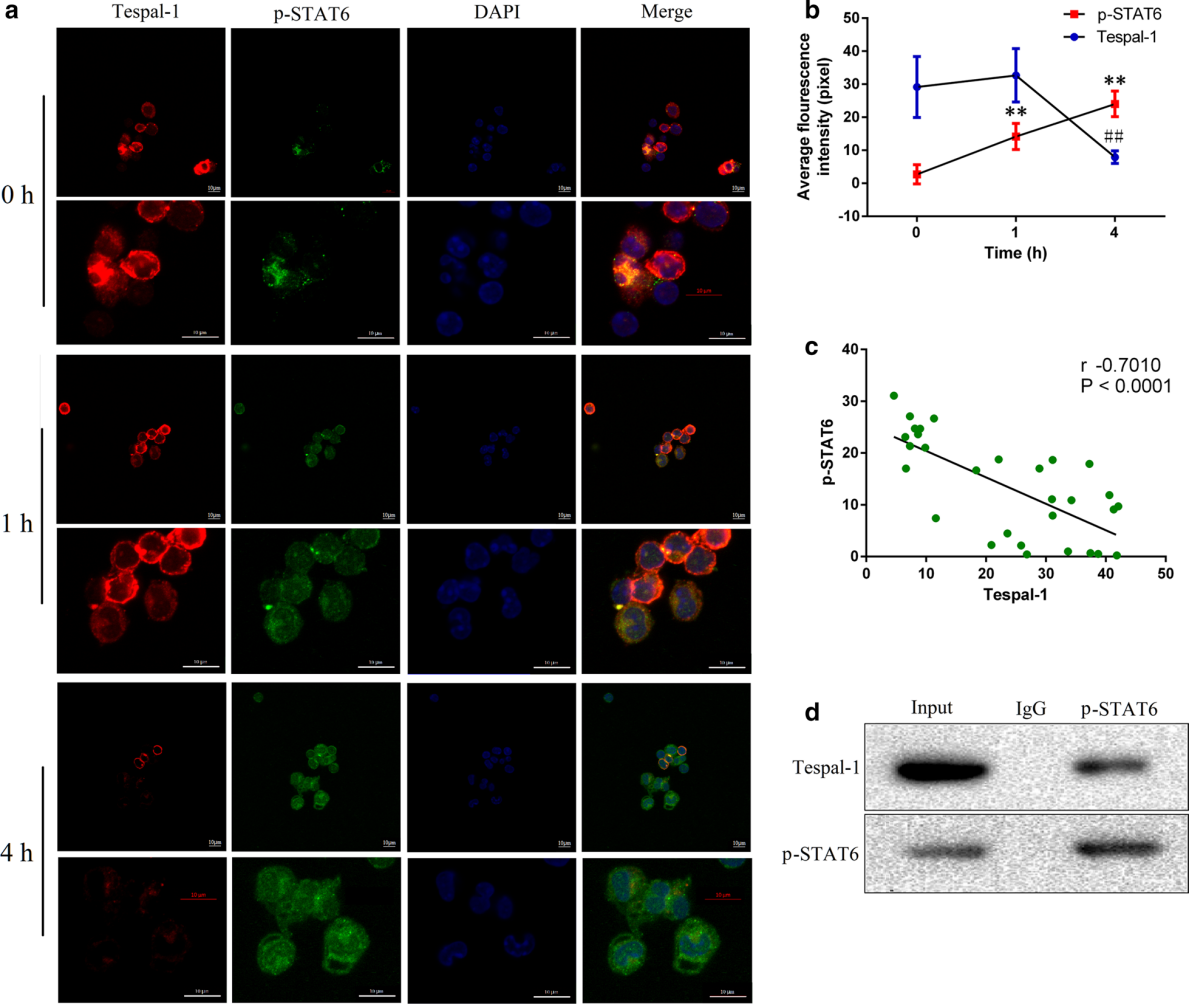
The relationship between Tespa1 and p-STAT6 detected by immunofluorescence and co-immunoprecipitation. Primary pulmonary mast cells were derived from the lungs of WT mice, and IL-4 (20 ng/mL) was simultaneously applied for 1 and 4 h. The expression and correlation of Tespa1 and p-STAT6 were detected by immunofluorescence. The relationship between Tespa1and p-STAT6 was detected by co-immunoprecipitation. **a** Immunofluorescence staining for Tespa1 and p-STAT6 after IL-4 stimulation. **b** Table of average Tespa1 and p-STAT6 fluorescence intensities. **c** Correlation analysis of Tespa1 and p-STAT6 expression of **a**. **d** Co-immunoprecipitation analysis of the relationship between Tespa1 and p-STAT6 in mast cells. **P < 0.01 compared with p-STAT6 at 0 min; ^##^*P* < 0.01 compared with Tespa1 at 0 min. Mean ± SD, *n* = 10

To investigate the role of *Tespa1* on IL4/STAT6 pathway in mast cells, we stimulated lung mast cells with IL-4 in vitro. Results showed that tryptase expression significantly increased in *Tespa1*^−/−^ mice than in the WT after IL-4 stimulation (Fig. 5). In order to further examine the potential mechanisms underlying these processes, primary pulmonary mast cells were derived from the lungs of WT mice, and IL-4 (20 ng/mL) was simultaneously applied for 1 and 4 h. The expression and correlation of Tespa1 and p-STAT6 were detected by immunofluorescence. The co-expression results of p-STAT6 and Tespa1 showed that IL-4 stimulated the increased and decreased expressions of p-STAT6 and Tespa1, respectively (Fig. 6a, b). In addition, a negative correlation was observed between Tespa1 and p-STAT6 in mast cells, as proven by the correlation analysis (*r* = − 0.7010, Fig. 6c). Furthermore, the relationship between Tespa1and p-STAT6 was detected by co-immunoprecipitation, and the results demonstrated an association between p-STAT6 and Tespa1 (Fig. 6d).

## Discussion

Tespa1 plays an important role in T cell development and negatively regulates Fc εR1-mediated mast cell activation and allergic reactions [13, 14]. The regulation of Tespa1 on mast cells is related to the negative regulation of the IL-4/STAT6 signaling pathway. This study provides a new perspective into the mechanism of asthma progression.

The analysis of Tespa1 mRNA expression in clinical samples including normal individual and asthmatic patients, showed that Tespa1 gene expression was lower, and IgE levels are higher in asthmatic patients, suggesting a relationship between Tespa1 and asthma. However, in this study, due to technical reasons, protein Tespa1 in sputum was not detected, which needs to be further discussed in future studies.

*Tespa1*^−/−^ mice were used for further in vivo experiments to extensively study the relationship between Tespa1 and the incidence of asthma. Results showed that *Tespa1*^−/−^ mice experienced more evident OVA-induced asthma attacks, which were manifested as increased airway resistance, and increased goblet cells. There was no significant difference in inflammation score between *Tespa1*^*−/−*^ OVA mice and WT OVA mice. Although the average score of *Tespa1*^*−/−*^ OVA mice was higher than that of WT OVA mice, it indicated that Tespa1 had little effect on the degree of inflammation during asthma attack. Toluidine blue staining showed that KO *Tespa1* gene could increase the number of mast cells. Immunohistochemistry indicated that the expression of tryptase, a marker of mast cell activity [32, 33], is more reactive in *Tespa1*^−/−^ asthmatic lungs than in WT mice, suggesting that Tespa1 could negatively regulate the aggregation and activation of mast cells. Hence, Tespa1 is associated with the proliferation and activation of mast cells in asthmatic mice. To further determine the role of Tespa1 in mast cells, we performed in vitro studies and observed that tryptase activation induced by IL-4 in *Tespa1*^−/−^ mast cells was evidently enhanced compared with in WT cells, and its peak activation time was consistent, all at 4 h after stimulation [34, 35], indicating that Tespa1 caused a certain inhibitory effect on mast cell activity.

The increased number and activity of mast cells mainly cause asthma, which involves many signaling pathways, in which the role of IL-4/STAT6 pathway in mast high sensitivity has been studied [36, 37]. Moreover *STAT6*^−/−^ and *IL-4*^−/−^ mice yield similar phenotypes, and cannot induce Th2 production, nor produce normal amounts of IgE, or even CD23, and IL-4 receptor subunit alpha [38–40]. Moreover, the hyperactivity of the IL-4/STAT6 pathway is associated with asthma and chronic obstructive pulmonary disease, and the transcriptional inhibitors of this pathway are potential targets for the prevention and treatment of diseases caused by the hyperactivity of the IL-4/STAT6 pathway [41, 42].

To study the effect of Tespa1 on STAT6 pathway, we detected the changes in IL-4 and IL-13 in the BAL of asthmatic mouse model, and the results showed that IL-4 and IL-13 in *Tespa1*^−/−^ mice significantly increased compared with those in the WT asthma group. And level of p-STAT6 expression also increased, further indicating the negative regulatory effect of Tespa1 on the STAT6 signaling pathway. The STAT6 signaling pathway can be regulated by IL-4/JAK1 and IL13/JAK2 [36, 43]. Western blot analysis results of lung tissues showed that in *Tespa1*^−/−^ asthma mice, the phosphorylation level of JAK1 increased, but the increase in P-JAK2 level was negligible, indicating that *Tespa1* mainly regulates the IL-4/JAK1 signaling pathway. Experiments showed that tryptase expression increased in *Tespa1*^−/−^ mast cell compared to with that WT in vitro, indicating that Tespa1 could negatively regulate the activation of mast cells. In addition, after IL-4 stimulation, the expression of p-STAT6 increased, whereas that of Tespa1 decreased. Thus, a negative correlation exists between p-STAT6 and Tespa1. After activation, p-JAK1 and p-JAK2 aggregate with STAT6 in the cytoplasm, phosphorylating Tyr or Ser at C-terminal of STAT6. p-STAT6 then forms a dimer that enters the nucleus, binds to the promoter, and initiates transcription and expression of the corresponding genes such as IL-4 and IL-13 [44]. The immunofluorescence results of this study showed that Tespa1 was dotted in the cytoplasm and co-localized with p-STAT6, indicating that the regulation of Tespa1 on p-STAT6 mainly occurred in the cytoplasm, but had no effect on the p-STAT6 entering the nucleus. When further exploring the relationship between Tespa1 and STAT6, co-immunoprecipitation results showed that Tespa1 and STAT6 directly interacted with each other.

## Conclusion

In this study, experiments have confirmed that Tespa1 negatively regulates mast cells during asthma, and this event is related to the IL-4/STAT6 signaling pathway. Thus, Tespa1 may be a potential target for asthma prevention and treatment. However, two deficiencies were identified. First, the exact mechanism of Tespa1 in regulating STAT6 still needs further study. Second, Tespa1 expression was also weakened after STAT6 signal activation. Hence, whether a cross-talk network exists between Tespa1 and IL-4/STAT6 signaling pathway is unclear.

## Data Availability

The datasets used and/or analyzed during the current study are available from the corresponding author on reasonable request.
